# Menstrual Tampons Are Reliable and Acceptable Tools to Self-Collect Vaginal Microbiome Samples

**DOI:** 10.3390/ijms241814121

**Published:** 2023-09-15

**Authors:** Florence Turner, Josephine Drury, Dharani K. Hapangama, Nicola Tempest

**Affiliations:** 1 Centre for Women’s Health Research, Department of Women’s and Children’s Health, Institute of Life Course and Medical Sciences, University of Liverpool, Member of Liverpool Health Partners, Liverpool L8 7SS, UK; f.turner2@student.liverpool.ac.uk (F.T.); jadrury@liverpool.ac.uk (J.D.); dharani@liverpool.ac.uk (D.K.H.); 2 Liverpool Women’s NHS Foundation Trust, Member of Liverpool Health Partners, Liverpool L8 7SS, UK; 3 Hewitt Centre for Reproductive Medicine, Liverpool Women’s NHS Foundation Trust, Liverpool L8 7SS, UK

**Keywords:** vaginal microbiome, tampon, vaginal swab

## Abstract

Many women report embarrassment as the cause for their avoidance of routine gynaecological screening appointments. Methods of self-collection of bio samples would perhaps encourage women to participate in routine screening programs. The vaginal microbiome plays a key role in women’s health and reproductive function. Microbial disturbances can result in the loss of lactobacillus dominance, also known as dysbiosis, associated with an increased risk of contracting sexually transmitted infections (STIs), pregnancy complications and infertility. Our primary aim was to determine if vaginal microbiome screening results are comparable between two methods for self-collected sample acquisition: tampons and lower vaginal swabs (LVSs). Secondary aims included the assessment of the effect of pre-analytic storage on the data (to streamline processing), the prevalence of dysbiosis and the acceptability of the tampons to the participants. Statistical analysis revealed no significant difference in the microbiome data, from tampons versus LVSs or fresh versus frozen samples. The prevalence of dysbiosis in this population of healthy volunteers was 42.9%. The questionnaire data revealed that 52.4% of volunteers use tampons every period, and the majority of volunteers rated the tampons as 5 on a 1–5 Likert scale regarding their perceived comfort using tampons. All (100%) of volunteers were happy to provide a tampon as a sample for testing. The findings from this study show that tampons and LVSs were comparable when analysing the vaginal microbiome, with potential superiority of the tampon with regard to patient acceptability. Self-collection of vaginal secretions for gynaecological screening using tampons warrants further research as this could change the screening landscape, ensuring wider participation and increasing efficacy.

## 1. Introduction

A major barrier to improving female genitourinary health in the United Kingdom (UK) is the number of missed routine appointments. The latest data for the National Health Service (NHS) cervical screening programme 2021–2022 showed only 69.9% of eligible individuals were adequately screened [1]. A recent survey revealing that a quarter of young women failed to attend screening due to embarrassment [2]. Self-collection devices may reduce their embarrassment, and thus, they are urgently needed to encourage women to engage fully in screening programs such as those for human papilloma virus (HPV), sexually transmitted infections (STIs) and vaginal dysbiosis, since these can be undertaken from the comfort of their own home [3,4].

The composition of the vaginal microbiome plays a significant role in women’s health and the development of pathological conditions [5]. The vaginal microbiome is dominated by lactobacilli species, which create a protective environment through the production of lactic acid, lowering the vaginal pH to <4.5 [6]. Lactic acid has been shown to have microbicidal and virucidal properties, helping to protect from infection [7]. Microbial imbalances can lead to an environment susceptible to the overgrowth of opportunistic bacteria resulting in loss of lactobacillus dominance, dysbiosis [8], which had a prevalence of 38.5% in a cohort of women living in Amsterdam [9]. Lack of lactobacillus can lead to a negative impact on women’s health and reproductive function including the increased risk of acquiring STIs [10,11], suffering with infertility [12] and developing pregnancy complications [13].

The development of new technology has enabled rapid analysis of the vaginal microbiome with ease using quantitative real-time polymerase chain reaction (qPCR) [14]. The vaginal microbiome is not routinely assessed but in certain research settings and private scenarios, women can access the test, which is usually undertaken through a vaginal swab [14]. 

Several collection devices can be used to collect specimens from the female genital tract, yet no consensus exists on a universally accepted best routine device [15,16]. Recent studies have utilised menstrual tampons as a biospecimen collection device for high-risk HPV messenger ribonucleic acid (mRNA), deoxyribonucleic acid (DNA) and endometrial cancer detection, comparing these results to endocervical swabs and revealing no difference in test positivity rates [17,18,19]. Most women are familiar with tampons and their use as a clinical test could potentially encourage previously embarrassed or scared women to seek and accept the associated preventative or screening healthcare interventions. 

In this study, we aimed to determine if the results of vaginal microbiomes obtained via two self-collection methods, tampons and lower vaginal swabs (LVSs), are comparable. The secondary aims of our study included determining if the samples can be stored prior to processing without altering the microbiome data, the prevalence of dysbiosis and assessing if tampons are an acceptable self-collection tool for women. 

## 2. Results

A total of 21 healthy volunteer women aged 21–39 years gave informed written consent and were recruited to participate in the study. Each volunteer provided two samples that were analysed separately as fresh and frozen tampon, and fresh and frozen LVS samples. The initial analysis of the samples included sample intake control (SIC), total bacterial mass (TBM) and presence of contamination. Out of the 84 samples, 80 (95.2%) had sufficient material for diagnostic analysis (TBM > 10^6.0^). This left four (4.8%) insufficient samples, one of which (1.2%) was detected as having no material and the remaining three (3.6%) samples with a TBM range from 10^5.0^–10^5.7^ (Table 1). Contamination was detected in three (3.6%) samples. 

### 2.1. Tampon versus LVS

**Figure 1:**
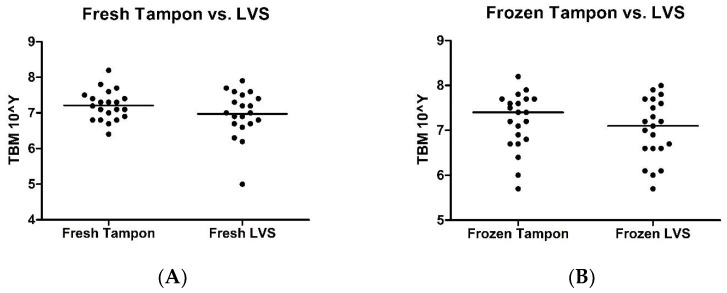
Scatter plots illustrating total bacterial mass comparison between samples collected via tampons versus samples collected via lower vaginal swabs. (**A**) Samples processed immediately. (**B**) Samples frozen at −20 °C for 2–4 weeks.

Using the Wilcoxon signed-rank test, no significant differences were identified in TBM between fresh tampons versus fresh LVSs (*p* = 0.295; Figure 1A) and frozen tampons versus frozen LVSs (*p* = 0.370; Figure 1B).

### 2.2. Fresh versus Frozen

**Figure 2:**
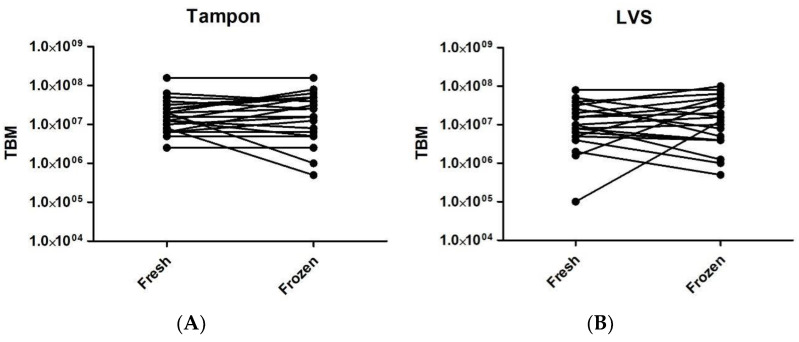
Before and after graphs illustrating sample total bacterial mass in immediately processed samples (fresh) compared to samples frozen for 2–4 weeks at −20 °C before being processed (frozen). (**A**) Tampon samples processed immediately versus tampon samples processed following freezing. (**B**) LVS samples processed immediately versus LVS samples processed following freezing.

The median TBM for fresh tampons, frozen tampons, fresh LVSs and frozen LVSs were 10^7.2^, 10^7.4^, 10^7.0^, and 10^7.1^, respectively (Figure 2A,B), showing no significant differences. 

### 2.3. Staphylococcus spp.

Staphylococcus spp. were identified in 20 (95.2%) volunteers; interestingly, in 3 of these volunteers (15%), detection was only by LVS. 

### 2.4. Diagnostic Conclusions

The Femoflor^®^ 16 RealTime_PCR v7.9 DNA-Technology software determined diagnostic conclusions for 78 of the 84 samples (92.9%) (Table 2). No conclusion was obtained for two frozen samples, despite both having adequate TBM. 

Of the 21 volunteers, 12 (57.1%) were concluded from all valid samples to have had normocenosis. Two (9.5%) volunteers had an overall picture of apparent anaerobic dysbiosis (AAnD). Numerous samples had a combination of conclusions (Table 2). Interestingly VMF8, VMF12 and VMF13 all had identical diagnoses with tampon samples concluded as moderate anaerobic dysbiosis (MAnD) and LVS samples concluded as AAnD.

### 2.5. Tampon Acceptability

**Figure 3:**
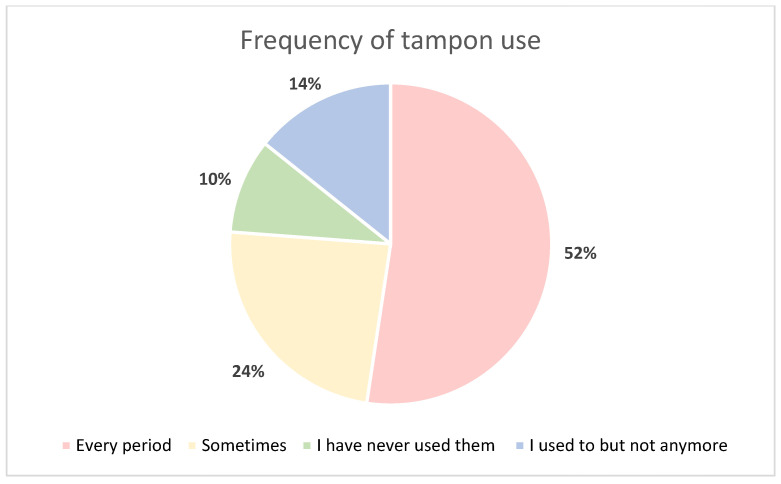
Pie chart illustrating volunteer questionnaire response to ‘How often do you use tampons?’.

**Figure 4:**
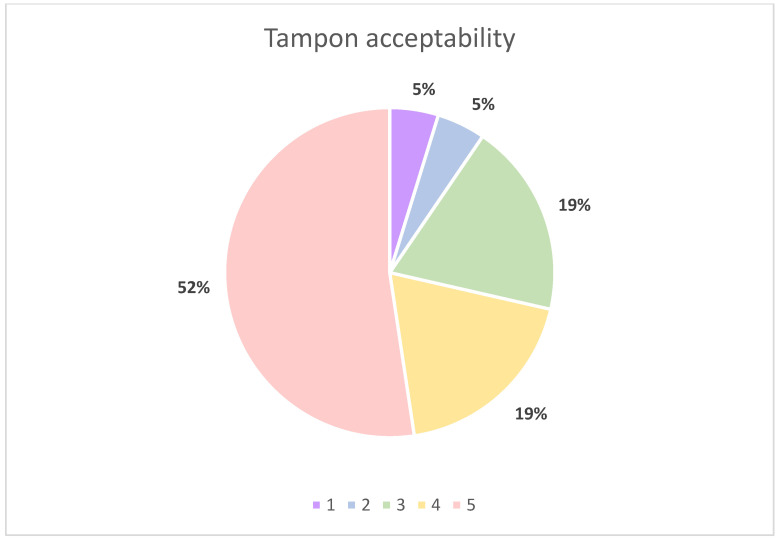
Pie chart illustrating volunteer questionnaire responses to ‘how do you feel about using tampons?’; 1 = ‘I find the process pretty uncomfortable and want to get it over with’ and 5 = ’It doesn’t bother me, it’s natural’.

All volunteers, 21 (100%), completed the questionnaire. The majority of volunteers stated that they use tampons every period (Figure 3). Volunteers were asked to rate the use of the tampon on a 5-point Likert scale, 1 being ‘I find the process pretty uncomfortable and want to get it over with’ and 5 being ‘It doesn’t bother me, it’s natural’ with no specific definitions beyond the two extremes of the scale. Out of the 21 volunteers, 15 (71.4%) gave favourable answers for tampon use (Figure 4). Finally, 100% of volunteers stated they would be happy to provide a tampon as a diagnostic test.

## 3. Discussion

This is the first study to comparatively assess menstrual tampons versus LVSs for the analysis of the vaginal microbiome in a group of healthy female volunteers. 

Statistical analysis of TBM between tampon and LVS samples revealed no significant difference, suggesting tampons are comparable collection devices when compared to LVS which are currently and frequently used for vaginal microbiome analysis.

No statistically significant differences were observed between corresponding fresh and frozen samples, allowing the conclusion that samples can be frozen prior to processing. Previous studies reported samples for vaginal microbiome analysis have been processed immediately after collection [14], but this obviously causes challenges in the research and clinical setting. The ability to freeze samples before processing allows flexibility in sample collection, batch processing and thus, will result in cost savings. 

The analysis of the questionnaire data revealed that the majority of volunteers use tampons on a regular basis and 100% of volunteers stated that they would be happy to provide a tampon sample for clinical use. Therefore, these data suggest that tampons were well accepted by volunteers in this study as a collection device.

Previous studies have differed in regard to the usefulness of tampons as a collection device in different settings for different conditions. Menstrual tampons were shown to be superior when compared to endocervical swabs, self-collected swabs and urine specimens in one manuscript, which used PCR to diagnose both Neisseria gonorrhoea and Chlamydia trachomatis [20]. Meanwhile, HPV testing in self-collected tampons and swabs versus clinician collected samples reported that all clinician specimens were sufficient, 27% of tampon samples were insufficient and 2% of self-collected swabs were insufficient [21]. These results are in contrast to our study, where only 2.4% of tampon samples were insufficient and a higher level of insufficiency was seen in our LVS samples (7.1%). Multiple clinical and logistical differences could have led to these differing results; fundamentally, we were collecting data investigating different conditions. Our tampons and swabs were collected to be processed straightaway or to be frozen, in comparison with the self-collected samples in the previous study [21], where samples were mailed in by participants. Those self-collected samples would therefore have been processed days after collection unlike the clinician collected samples in their study. In our study, the volunteers were given clear instructions to insert tampons for exactly 20 min, but in McLarty et al., the participants were instructed to insert tampons for a minimum of 2 h [21]. These methodological differences are expected to explain the different results observed.

Our questionnaire agrees with previous acceptability work. A UK study that assessed the acceptability of tampon sampling for detecting STIs in sex workers concluded that 95% of those who were questioned preferred self-sampling via tampons in comparison to clinician-collected conventional swabs [22]. Tampons may not be suitable for all people though, and importantly, regular tampon use seems to vary between ethnicities. A US study assessing tampon use in ethnic groups identified European American women as significantly more likely to use tampons in adolescence (71%) than African American women (29%) [23]. 

Further analysis of samples revealed the presence of Staphylococcus spp. in all except one volunteer. Three volunteers were only detected as having Staphylococcus spp. by LVSs. Staphylococcus spp. are known skin commensals [24]. A French study identified a greater frequency of S. aureus detection in tampons without applicators versus tampons with applicators [25], as used in our study. It is possible that when performing an LVS there is a greater chance of sampling from areas other than the intended site, hence misrepresenting the vaginal microbiota, than when using a tampon inserted via applicator. Selection of biospecimen collection device may be relevant to the accuracy of microbiome data and thus, researchers may need to ensure that they are using a device that ensures they are only sampling the intended areas relevant to their studies. 

Approximately 42.9% of self-collected samples from volunteers were identified as have some degree of dysbiosis; this is similar to existing larger studies showing dysbiosis in vaginal swab samples obtained by clinicians from a general population of volunteers [9]. We recruited 21 healthy female volunteers and it was not a primary aim of our study, so our data cannot be extrapolated to the general population, women with menstrual disorders or those women who are post-menopausal. In order to comment on the prevalence of dysbiosis in the general population, further research with a larger more representative study population would be needed and further studies assessing suitability of self-collected tampons with an analysis of factors including ethnicity, day of menstrual cycle and menopausal status would be necessary to establish tampons as a more universally suitable collection device. 

Our data have proven that a menstrual tampon is sensitive, specific and acceptable collection device for vaginal microbiome studies. Tampons, as a collection device, could encourage women to self-collect vaginal bio-samples for testing and screening of multiple conditions that currently require appointments with a trained medical professional. Since we know that many women are reluctant to seek STI testing and cervical smear tests, a seemingly more acceptable, self-collected menstrual tampon could eliminate embarrassment-associated avoidance, be time efficient and also be cost effective for health care providers. Furthermore, they may be used to potentially test for multiple conditions with increased participation and efficacy. 

## 4. Materials and Methods

### 4.1. Recruitment

The University of Liverpool Central University Research Ethics Committee for Physical Interventions approved the application (5347 Comparing two self-administered methods (a Daye tampon (London, UK) and a low vaginal swab (HydraFlock^®^ Standard Tip, Flexible Shaft, mwe Medical Wire, Corsham, UK)) to collected information on the vaginal microflora from healthy volunteers, on 10 October 2019). A total of 21 female volunteers (mean age 29 years) were provided with an information sheet before being asked to sign a consent form to participate in the study. Each volunteer was asked to wear a tampon for 20 min followed by performing 2 lower vaginal swabs and completing a questionnaire. The Daye tampon was placed immediately into a Universal tube (Starlabs, Milton Keynes, UK) and the LVSs were stored in the sterile sample tube they are manufactured in. The swabs and tampon were transported directly to the laboratory (within 30 min).

### 4.2. Questionnaire

The questionnaire consisted of open and closed questions as well as a 5-point Likert-style scale to assess tampon acceptability.

### 4.3. Sample Collection and Processing

**Figure 5:**
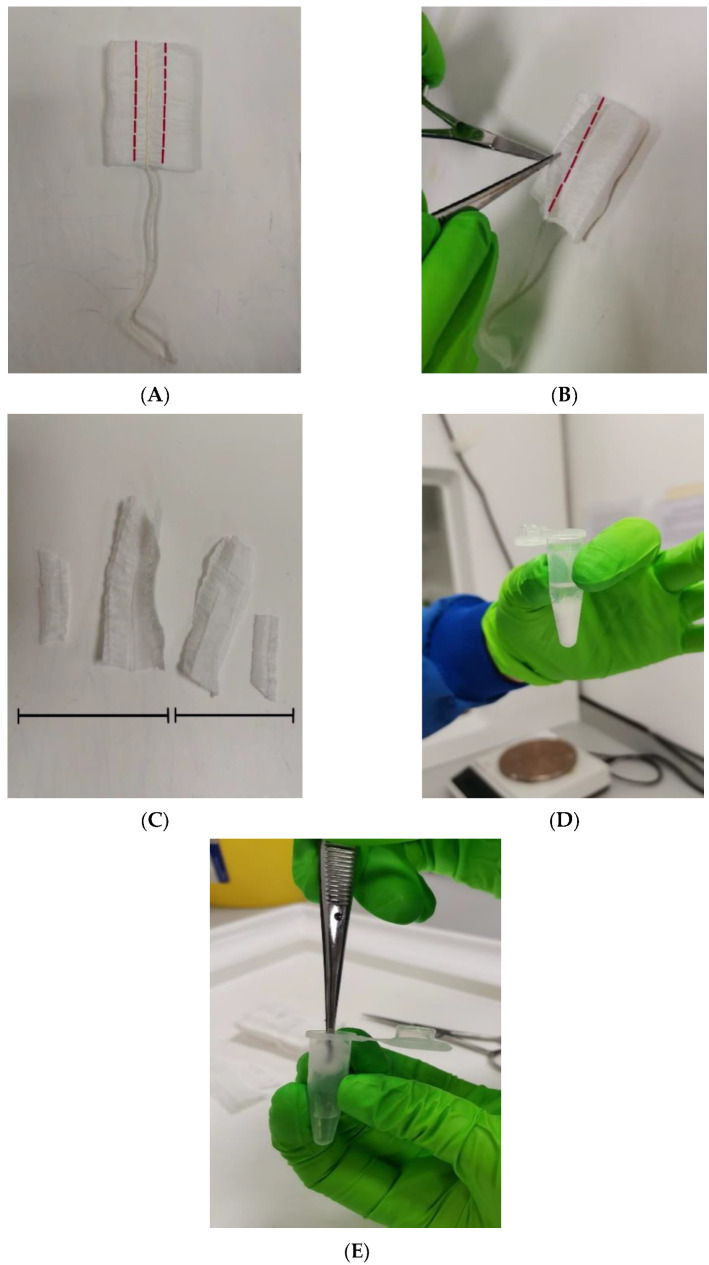
(**A**) The front of the tampon showing incisions made 0.5 cm from the midline. (**B**) The reverse side of the tampon showing the incision made to remove the tab from the midline. (**C**) Four pieces overall were utilised; two were frozen and two were processed immediately. (**D**) The tampon sample submerged in sterile PBS. (**E**). Forceps used to push tampon sleeve against side of tube to extract as much liquid as possible.

The tampon was removed from the Universal tube and placed in a class II safety cabinet. Sterile forceps and scissors were used to remove the tampon’s outer skin and a cut was made 0.5 cm on either side of the midline of the tampon bilaterally. The tab on the midline was also cut off and divided into two equal parts: one half was for immediate processing and the other half for freezing (Figure 5). The half for freezing was placed into a dry centrifuge tube and the half for immediate processing was placed into a 1.5 mL micro-centrifuge tube containing 500 µL sterile phosphate-buffered saline (PBS) pH 7.4 (Sigma Aldrich, Gillingham, UK). The tampon sleeve was soaked in the PBS and forceps were used to push the tampon sample against the side of the centrifuge tube to ensure that as much liquid as possible was extracted back into the bottom of the tube. Next, one of the swabs was removed from the sample tube and placed in a sterile dry 1.5 mL centrifuge tube for freezing and the remaining swab was agitated in a 1.5 mL micro-centrifuge tube containing 500 µL sterile pH 7.4 PBS for 1 min. A further 1.5 mL micro-centrifuge tube containing 500 µL sterile pH 7.4 PBS was used as a negative control. Frozen samples were stored at −20 °C for 2–4 weeks. When ready to be processed, frozen samples were removed from the freezer and allowed to thaw for 5 min before undergoing the steps above.

### 4.4. DNA Extraction 

DNA was extracted from the tampon sleeve, LVS and negative control tubes for each volunteer using a PREP-NA-PLUS Kit (DNA Technology, Moscow, Russia) according to the manufacturer’s instructions. In brief, the tubes were centrifuged for 10 min at 16,000× *g* before discarding the supernatant, leaving 100 µL of liquid in each tube. A 300 µL volume of lysis buffer, warmed to 65 °C, was added to each sample before they were briefly vortexed. The samples were incubated at 65 °C for 15 min before centrifugation for 30 s at 13,000× *g*. A 400 µL volume of precipitation buffer was added to each sample followed by a brief vortex and centrifuge for 15 min at 13,000× *g*. All supernatant was removed from the pellets and 500 µL of wash out solution 1 was added to each sample. The samples were inverted 5 times followed by centrifugation for 5 min at 13,000× *g*. All supernatant was again removed from the pellets, 300 µL of wash out solution 2 was added and the samples were inverted 5 times and centrifuged for 5 min at 13,000× *g*. Again, all supernatant was removed, and each sample was incubated at 65 °C for 5 min with lids open. A 300 µL volume of dilution buffer was added before a quick centrifuge and incubation at 65 °C for 10 min, a brief vortex, and then centrifuged for 30 s at 13,000× *g*. 

### 4.5. Femoflor^®^ 16 Real-Time PCR

**Figure 6:**
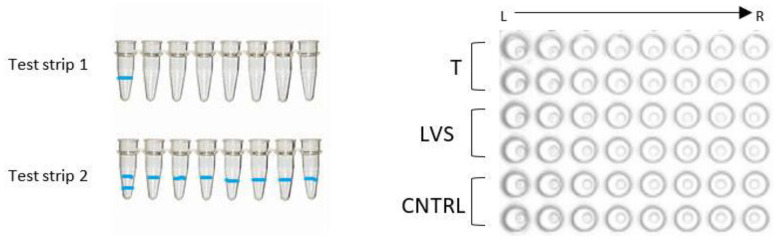
The two different types of test strips can be identified via the blue banding at the bottom of the tubes. Test strip 1 with a single blue line on the first tube from left to right and test strip 2 with two blue lines on the first tube from left to right. These are inserted into the PCR machine in the exact order as shown on the right, ensuring the correct orientation from left to right using the blue strips as reference.

The Femoflor^®^ 16 REAL-TIME PCR Detection Kit (DNA-Technology LLC, Moscow, Russia) was used following the manufacturer’s instructions. In short, two eight-tube strips were used for each sample (Figure 6). A 20 μL volume of mineral oil, 10 μL of taq polymerase and 5 μL of sample were added to each tube in the strips and briefly centrifuged prior to placing in the qPCR machine [25].

### 4.6. Determination of Diagnostic Conclusions

**Figure 7:**
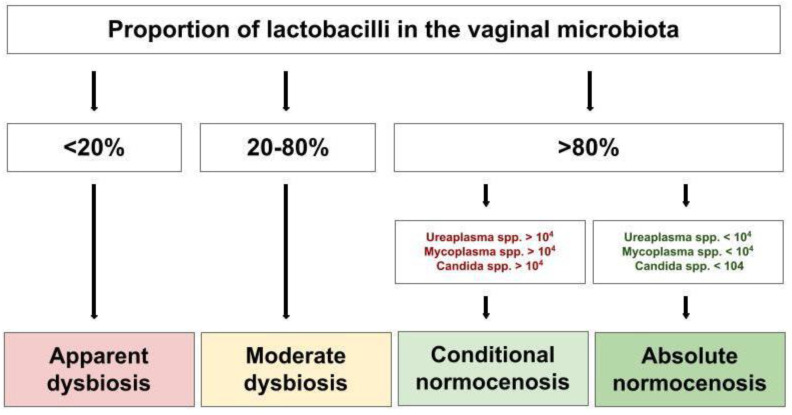
Illustration of how conclusions were determined using the Femoflor^®^ 16 programme. General conclusion determined by proportion of lactobacilli present in the sample population. Degrees of dysbiosis further categorised based on proportion of obligate and facultative anaerobes.

Real-Time_PCR v7.9 DNA-Technology was able to broadly categorise the samples by the proportion of lactobacilli in the vaginal microbial community with Normocenosis > 80% and Dysbiosis < 80%. Normocenosis is further categorised into absolute normocenosis (AN) and conventional normocenosis (CN) based on the quantity of Ureaplasma, Mycoplasma and Candida spp. (Figure 7). Dysbiosis can be further categorised based on lactobacilli proportion into moderate dysbiosis (MD; 20–80%) and apparent dysbiosis (<20%). Within these categories, there are three possible diagnoses, determined by comparing the proportion of facultative anaerobes to obligate anaerobes. Aerobic dysbiosis is concluded when the proportion of facultative anaerobes is more than 10% and the proportion of obligate anaerobes is less than 10% of the total bacterial load (TBL). Anaerobic dysbiosis is when the proportion of facultative anaerobes is less than 10% and the proportion of obligate anaerobes is more than 10% of the TBL. Mixed dysbiosis is when the proportion of facultative anaerobes is more than 10% and the proportion of obligate anaerobes is more than 10% of the TBL. The diagnostic sensitivity and specificity of Femoflor^®^ are 97% and 97%, respectively [25]. 

### 4.7. Data Interpretation and Analysis 

The samples were initially assessed by looking at the logarithmic values of SIC, TBM and the diagnosis concluded by the Femoflor^®^ 16 RealTime_PCR v7.9 DNA-Technology software (sensitivity 97%, specificity 97%). Negative controls were assessed to ensure that there were no microorganisms detected in the sample. For a sufficient sample, SIC > 10^4^ and TBM > 10^6^ were needed.

Statistical analysis was performed using GraphPad PRISM 5 software, Microsoft Excel 2019 and IBM SPSS Statistics 27. Wilcoxon signed-rank test was performed to determine any significant differences between matched pairs. Statistical significance was identified when *p* < 0.05. 

Anonymous questionnaire data were collated and analysed using Microsoft Excel 2019. 

## Figures and Tables

**Table 1:** The total bacterial mass (TBM) of the corresponding four samples for each of the volunteers. Highlighted boxes for VMF04, VMF10 and VMF12 identify samples with insufficient TBM for a valid analysis (minimum TBM required >10^6.0^).

Sample ID	Fresh Tampon	Frozen Tampon	Fresh LVS	Frozen LVS
VMF01	10^6.8^	10^6.8^	10^6.7^	10^6.6^
VMF02	10^7.4^	10^7.8^	10^7.7^	10^7.2^
VMF03	10^7.3^	10^7.4^	10^6.9^	10^7.0^
VMF04	10^6.8^	10^7.5^	10^5.0^	10^7.1^
VMF05	10^8.2^	10^8.2^	10^7.5^	10^8.0^
VMF06	10^7.6^	10^7.4^	10^6.7^	10^7.7^
VMF07	10^7.1^	10^7.7^	10^7.0^	10^6.1^
VMF08	10^7.1^	10^6.7^	10^7.4^	10^6.9^
VMF09	10^6.8^	10^7.1^	10^6.6^	10^6.0^
VMF10	10^6.9^	10^5.7^	10^6.3^	10^5.7^
VMF11	10^7.3^	10^6.0^	10^6.2^	10^7.6^
VMF12	10^7.5^	10^7.7^	10^7.2^	10^7.5^
VMF13	10^7.8^	10^7.6^	10^7.6^	10^7.8^
VMF14	10^7.0^	10^7.2^	10^7.3^	10^7.7^
VMF15	10^7.1^	10^6.9^	10^7.0^	10^7.2^
VMF16	10^7.7^	10^7.6^	10^6.8^	10^6.6^
VMF17	10^7.4^	10^7.7^	10^7.9^	10^7.9^
VMF18	10^6.7^	10^6.7^	10^6.9^	10^6.6^
VMF19	10^7.2^	10^7.2^	10^7.2^	10^7.3^
VMF20	10^6.4^	10^6.4^	-	10^6.1^
VMF21	10^7.3^	10^7.9^	10^7.6^	10^6.7^

**Table 2:** Summary of diagnostic conclusions calculated by the FEMOFLOR 16 RealTime_PCR programme for every sample from each volunteer. AN—absolute normocenosis, AAnD—apparent anaerobic dysbiosis, MAnD—moderate anaerobic dysbiosis, CN—conventional normocenosis, +c—candida spp. exceeding clinically significant threshold value, MMD—moderate mixed diagnosis.

Sample ID	Fresh Tampon	Frozen Tampon	Fresh LVS	Frozen LVS
VMF01	AAnD	AAnD	AAnD	AAnD
VMF02	AN	AN	AN	AN
VMF03	AN	AN	AN	AN
VMF04	AN	AN	-	AN
VMF05	MAnD	AN	MAnD	MAnD
VMF06	AN	AN	AN	AN
VMF07	AN	AN	AN	AN
VMF08	MAnD	MAnD	AAnD	AAnD
VMF09	AN	AN	AN	AN
VMF10	MAnD	-	CN +c	-
VMF11	AN	-	AN	AN
VMF12	MAnD	MAnD	AAnD	AAnD
VMF13	MAnD	MAnD	AAnD	AAnD
VMF14	AN	AN	AN	-
VMF15	CN	AN	MAnD	MAnD
VMF16	CN	CN	CN	CN
VMF17	AN	AN	AN	AN
VMF18	AN	N	AN	AN
VMF19	MMD	MAnD	AN	CN +c
VMF20	AAnD	AAnD	-	AAnD
VMF21	AN	AN	AN	AN

## Data Availability

The data presented in this study are available within this manuscript.
